# Clinical Characteristics for Differentiating Febrile Children With Suspected Kawasaki Disease Diagnosis

**DOI:** 10.3389/fped.2020.00221

**Published:** 2020-05-05

**Authors:** Jia-Huei Yan, Ling-Sai Chang, Yi-Ju Lin, Mindy Ming-Huey Guo, Ying-Hsien Huang, Ho-Chang Kuo

**Affiliations:** ^1^Department of Pediatrics, Kaohsiung Chang Gung Memorial Hospital and Chang Gung University College of Medicine, Kaohsiung, Taiwan; ^2^Kawasaki Disease Center, Kaohsiung Chang Gung Memorial Hospital, Kaohsiung, Taiwan; ^3^Department of Pediatrics, Chiayi Chang Gung Memorial Hospital, Chiayi, Taiwan

**Keywords:** clinical characteristics, febrile children, kawasaki disease, C-reactive protein, neutrophil-to-lymphocyte ratio

## Abstract

**Background:** Kawasaki disease (KD) is a form of vasculitis that primarily affects children under the age of 5 years old. Patients may be missed or diagnosis delayed when initial clinical symptoms do not fulfill the traditional criteria or a normal echocardiography was found. In this study, we aimed to analyze factors that clinicians could use to differentiate febrile children suspected of KD.

**Method:** We retrospectively enrolled in this study a total of 50 febrile children who were initially suspected of KD, but they did not meet the American Heart Association (AHA) criteria for a diagnosis. However, some of these patients were diagnosed with KD during their second visit. We analyzed patients' characteristics, clinical symptoms, and laboratory data (initial data in the first visit).

**Results:** In total, 50 patients were enrolled in the study. Of those, ten patients were diagnosed with KD on their second visit (group 1), while the other 40 patients still did not fit a KD diagnosis (group 2). A higher neutrophil-to-lymphocyte ratio (NLR, *p* = 0.037) and higher C-reactive protein levels (CRP, *p* = 0.02) were found in group 1 when compared to group 2. A patient with a NLR >1.33 combined with a CRP more than 33 mg/L was more likely to have KD (Sensitivity 90%, specificity 69.2%, *p* = 0.001; Odds ratio 20.25, 95% confident interval 2.3–178.25).

**Conclusion:** Among patients suspected of KD that did not initially meet the criteria, clinicians should pay special attention to elevated neutrophil-to-lymphocyte ratios and CRP levels and closely follow up such patients.

## Introduction

Kawasaki disease (KD) is characterized as a medium-sized vasculitis that particularly involves the coronary arteries. It is the most common cause of acquired heart disease in children under the age of 5 years old (1). The etiology of KD is still not completely known. The incidence of KD is higher in Asia than in the United States and Europe and has been increasing in recent decades (2, 3). In Taiwan and Japan, the incidence is 3 to 15 times higher than in North America (1). KD can lead to coronary artery anomalies (CAA) if not properly treated with intravenous immunoglobulin (IVIG). In the pre-IVIG era, the CAA incidence rate was 20–25%, but IVIG therapy has reduced that to 3–5% (1, 4). Delaying both diagnosis and treatment are major risk factors of CAA persistence and such adverse cardiac events as myocardial ischemia and sudden cardiac arrest (4). The importance of early differentiation of KD from other fever patients cannot be overlooked.

KD diagnostic criteria is currently based on clinical findings, echocardiogram, and AHA supplementary laboratory data, so that clinicians can exclude other similar diseases (1). However, KD diagnosis can often be missed or postponed in the case of atypical or incomplete clinical presentation. The characteristic clinical features of KD include prolonged fever that lasts more than 5 days, and four of the following five symptoms: oral changes, non-exudative conjunctivitis, skin rash, extremity changes, and cervical lymphadenopathy (1, 5). Nevertheless, these symptoms and signs may appear at different times during the febrile period, and the presentation of illness is often not apparent in infants younger than 6 months old or incomplete KD patients (1, 6, 7). Pyuria may be treated as a urinary tract infection while subsequent skin rash, injected eyes, or red lips may be considered a reaction to antibiotics. KD may cause retropharyngeal edema and KD shock syndrome, which are often mistaken as being bacterial in origin (8). In this study, we reviewed suspected KD patients that did not fulfill the criteria at their first clinic visit, comparing and differentiating their clinical findings regarding KD diagnosis between it and their second visit.

## Method

### Study Design

We retrospectively reviewed 50 children who were suspected of KD by pediatric clinicians, but whose clinical conditions did not meet the American Heart Association's (AHA) criteria for diagnosing typical KD or incomplete KD (1) on the first visit. The cases were divided into two groups based on whether they fit the KD diagnosis on the second visit. For this study, we examined patients that visited Kaohsiung Chang-Gung Memorial Hospital from November 2008 to September 2018, and patients without complete medical chart records were excluded.

The AHA criteria for typical KD diagnosis is based on fever ≥5 days and the presence of ≥4 of the 5 clinical features (as our rapid memory method 1-2-3-4-5) (5): oral changes (1 mouth) (strawberry tongue, erythematous or cracking of lip, and/or erythema of oral mucosa), non-exudative bilateral bulbar conjunctival injection (2 eyes), cervical lymphadenopathy (3 fingers to check neck lymph node ≥1.5 cm diameter, usually unilateral), 4 limbs extremity changes (erythema and edema of the hands and feet and/or periungual desquamation), and dysmorphism skin rash (5 means many rashes). Incomplete KD is defined as children with fever ≥5 days, CRP ≥ 30 mg/L, and/or ESR ≥ 40 mm/hour, as well as two or three compatible criteria plus a positive echocardiogram finding or three or more of six laboratory features (anemia by age, platelet count ≥450000/mm^3^ after 7th day of fever, albumin ≤ 3g/dL, elevated ALT level, WBC count ≥ 15000/mm^3^, urine ≥ 10 WBC/high power field). Based on the AHA recommendations for an incomplete KD diagnosis, an echocardiogram finding is considered positive if any of the following three conditions are met (1): left anterior descending (LAD) coronary artery or right coronary artery (RCA) Z score of ≥2.5; presence of a coronary artery aneurysm in the echocardiogram; or the presence of ≥3 other suggestive features, including decreased left ventricular function, mitral regurgitation, pericardial effusion, or Z scores in LAD coronary artery or RCA of 2 to 2.5. This study was approved the Institutional Review Board of Chang Gung Memorial Hospital (102-0364B).

### Data Analysis

We analyzed the patients' characteristics, clinical symptoms, and laboratory data (initial data of first visit) (Tables 1, 2). Characteristics include patients' age, gender, and days of fever before their first visit. We compared clinical symptoms based on KD diagnostic criteria, including oral changes, non-exudative conjunctivitis, extremity changes, skin rash, and lymphadenopathy (>1.5 cm). We used the Kolmogorov-Smirnova test to examine the data and reveal non-normal distributions. We analyzed continuous variables using the Mann-Whitney test, while the chi-square test and Fisher's exact test were adopted for categorical variables. The cut-off point was determined through the ROC curve and Youden index. All data are presented by percentage and median with interquartile range (IQR). We considered *p* < 0.05 statistically significant. All statistical tests were performed using SPSS 22.0 (SPSS, Inc., Chicago, Illinois).

**Table 1:** Patients' characteristics and clinical symptoms and signs.

		**KD (Group 1)**	**Not KD (Group 2)**	***P*-value**
Total (*N*)		10	40	
Age [year; median (IQR)]		2.2 (1.5–4.3)	1.4 (0.8–3.2)	0.121
Gender	Male	5	27	0.463
	female	5	13	
Days of fever^*^ [median (IQR)]		4.5 (3–5)	4 (3–6)	0.555
Initial clinical symptoms and signs [*N* (%)]				
Oral change		7 (70%)	25 (62.5%)	0.73
Non-exudative conjunctivitis		8 (80%)	26 (65%)	0.468
Extremity change		4 (40%)	20 (50%)	0.728
Skin rash		5 (50%)	30 (75%)	0.143
Lymphadenopathy		1 (10%)	4 (10.3%)	1

* *Days of fever before first visit to our clinic*.

**Table 2-1:** Patients' laboratory data [median (IQR)] at first visit.

	**KD (Group 1)**	**Not KD (Group 2)**	***P*-value**
Total (*N*)	10	40	
WBC (1000/mm^3^)	11 (8.8–13)	11.5 (9–15.4)	0.594
Hemoglobulin(g/dL)	11.3 (10.7–12)	11.5 (11–12.2)	0.481
Platelet (1000/mm^3^)	297.5 (248.5–408.5)	313.5 (259.5–392.8)	0.799
Neutrophil (%)	63.7 (51.9–77)	53 (43–65)	0.051
Lymphocyte (%)	29.7 (14–32.7)	37.9 (25.2–49.9)	0.048^*^
Neutrophil to lymphocyte ratio	2.1 (1.5–5.5)	1.39 (0.85–2.6)	0.037^*^
CRP (mg/L)	62.3(35.7–99.9)	26 (8.3–48.9)	0.020^*^
GOT (IU/L)	33 (24–52)	35.5 (28–44)	0.617
GPT (IU/L)	21 (12–55.8)	22 (14.8–32)	0.949
Albumin (g/dL)	4.1 (3.8–4.2)	4.2 (4–4.4)	0.098
Urine WBC (/μL)	6 (0–29)	1 (0–3)	0.200

* *p < 0.05*.

**Table 2-2:** Patients' laboratory data [median (IQR)] at second visit.

	**KD (Group 1)**	**Not KD (Group 2)**	***P*-value**
Patient numbers	10/10	7/40	
WBC (1000/mm^3^)	11.1 (9.6–13.0)	9.6 (7.4–11.7)	0.299
Hemoglobulin (g/dL)	11.0 (10.0–11.7)	11.5 (10.7–12.2)	0.252
Platelet (1000/mm^3^)	544.0 (403.5–591.5)	484.0 (327.0–527.0)	0.299
Neutrophil (%)	55.1 (35.5–69.0)	36.8 (19.0–56.0)	0.299
Lymphocyte (%)	32.0 (20.8–59.0)	53.8 (25.0–66.0)	0.470
Neutrophil to lymphocyte ratio	1.67(0.59-3.55)	0.68(0.29–2.32)	0.408
CRP (mg/L)	10.3 (2.5–26.4)	1.1 (0.2–10.1)	0.070
GOT (IU/L)	33.0 (26.8–42.3)	32.0 (25.0–50.0)	0.887
GPT (IU/L)	14.0 (8.5–42.0)	21.0 (15.0–52.0)	0.417
Albumin (g/dL)	3.9 (3.8–4.4)	^¶^	
Urine WBC (/μL)	0 (0–0)	^¶¶^	

¶ *only 2 patients in group 2 obtained albumin (3.7, and 4.0 g/uL, respectively) in not KD group in second visit*.

¶¶ *only 1 patient obtained U-WBC data (61/uL) in not-KD group in second visit*.

* *p < 0.05*.

## Results

**Figure 1:**
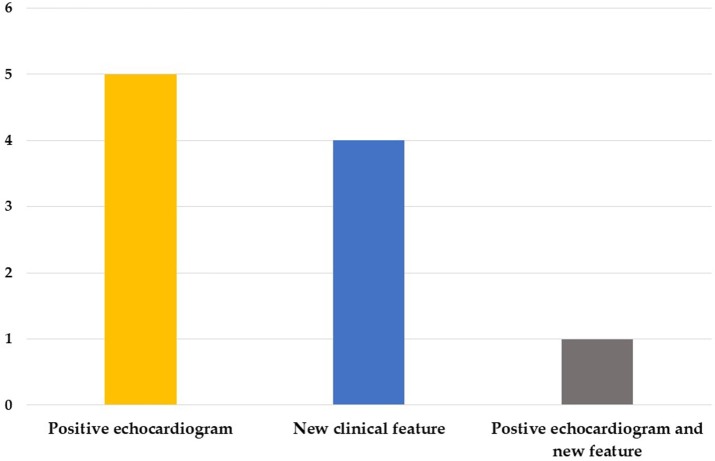
Factors for diagnosis of KD based on the second visit.

We enrolled 50 patients in this study, including 32 boys and 18 girls, 10 of which were diagnosed as KD with a positive echocardiogram finding or one or more new clinical features in their second visit (group 1) and 40 non-KD patients (group 2). The mean interval between first visit and second visit was 8.4 days. In group 1, five cases were diagnosed by a positive echocardiogram, four cases were diagnosed by new clinical features, and one case was diagnosed by both a positive echocardiogram and new features in the second visit (Figure 1); Of the 10 patients, 6 of them were atypical KD on the diagnosis in second visit. Moreover, in group 1, nine of them had normal echocardiography result and 1 did not perform echocardiography during 1st visit. However, 6 of them had coronary arteries dilatation or aneurysms formation at second visit. The mean ages were 2.2 (1.5–4.3) years and 1.4 (0.8–3.2) years old, and the days of fever before the first visit were 4.5 (3–5) days and 4 (3–6) days for group 1 and group 2, respectively. We found no statistically significant differences regarding age, gender, days of fever, and initial clinical symptoms between the two groups (Table 1).

We further analyzed laboratory data and found a higher neutrophil-to-lymphocyte ratio (NLR, *p* = 0.037) and higher C-reactive protein levels (CRP, *p* = 0.02) in group 1 compared to group 2 (Table 2). Cut-off points determined by ROC curve presented NLR > 1.33 (AUC = 0.715, sensitivity 100%, specificity 47.5%, *p* = 0.008, Odds ratio 1.48, 95% confident interval 1.16–1.88) and CRP > 33 mg/L (AUC = 0.74, sensitivity 90%, specificity 59%, *p* = 0.011, Odds ratio 12.94, 95% confident interval 1.49–112.44), both of which have a probability of predicting KD. By combining NLR > 1.33 and CRP > 33 mg/L, we found a higher odds ratio of 20.25 (95% confident interval 2.30–178.25) of KD predicting probability, as well as 90% sensitivity and 69.2% specificity (*P* = 0.001) (Table 3).

**Table 3:** The sensitivity, specificity and odds ratio of cut-points.

	**NLR > 1.33**	**CRP > 33 mg/dL**	**NLR > 1.33 plus CRP > 33 mg/dL**
Sensitivity	100%	90%	90%
Specificity	47.5%	59%	69.2%
*P*-value	0.008	0.011	0.001
Odds ratio (95% confident interval)	1.48 (1.16–1.88)	12.94 (1.49–112.44)	20.25 (2.30–178.25)

## Discussion

In this study, we demonstrated patients who were suspected of KD but did not meet either AHA diagnostic criteria. A diagnosis of KD consists of refractory and prolonged fever for more than 5 days and traditional four of five clinical presentation symptoms. However, patients with atypical or incomplete KD pose a challenge for pediatricians, particularly young infants <6 months old and older children at high risk of CAA development (1, 9). Atypical or incomplete presentation refers to patients with prolonged fever that only fit two or three clinical features. Symptoms may be dispersed over a period of time; for example, desquamation of the fingers and toes is a late finding that appears at 2~3 weeks after onset of fever (10). KD may be misdiagnosed without careful clinical observation or echocardiogram finding. The current treatment recommendation is administering IVIG therapy within 10 days of the onset of illness (1, 11), and postponed treatment is the leading cause of CAA formation (4, 7, 9–11). Clinicians should closely follow up patients who are suspected of KD but do not fit the criteria.

The neutrophil-to-lymphocyte ratio (NLR) is considered the absolute neutrophil count divided by the absolute lymphocyte count and is a simple and inexpensive test to perform. Neutrophils reflect ongoing inflammation and enhanced inflammatory mediator secretion; on the other hand, lymphocytes represent immune regulatory response (12). NLR is a vital biomarker of the balance between inflammation and immune regulation and has been studied with regard to the prognostic and risk factors of cardiovascular diseases and cancer (13–15). Furthermore, previous studies have also demonstrated its predictive value for KD. A higher NLR is associated with IVIG-resistant KD and CAA formation (16–19). Our study demonstrated that the cut-off value of NLR of 1.33 has an odds ratio of 1.48 to predict KD with high sensitivity. Such higher sensitivity can indicate that clinicians should follow up with patients more closely and help them make a real KD diagnosis earlier.

CRP levels are responsible for a patient's inflammation status and can serve as a differentiating factor in this study. According to AHA guidelines for incomplete KD diagnosis, CRP > 3.0 mg/dL (=30 mg/L) and/or ESR ≥ 40 mm/hour is considered supplementary laboratory data (1). We determined that the CRP cut-off point of more than 33 mg/L can also be a predicting factor of KD, with an odds ratio of 12.94 (95% confident interval 1.49–112.44, sensitivity 90%, specificity 59%, *p* = 0.011). However, CRP levels are elevated not only in the case of inflammation but also in patients with an infectious disease. Clinicians must cautiously interpret CRP levels and rule out the possibility of pathogen invasion and other systemic inflammation diseases, while a CRP level <33 mg/L is common in refractory febrile children with infection. Therefore, we combined the two cut-off values of both NLR and CRP to achieve a higher odds ratio of 20.25, a mildly lower sensitivity but better specificity than using NLR or CRP independently (95% confident interval 2.30–178.25, sensitivity 90% and specificity 69.2%, *P* = 0.001). For patients suspected of KD but that do not meet either of the AHA criteria for typical or incomplete KD, pediatric clinicians should pay particular attention to elevated NLR and CRP levels and closely follow up with those patients. As mentioned in the Table 1, the febrile days before 1st visit ranged from 3 to 6 days. It meant that we should pay attention and check NLR and CRP when we raise the suspicion to KD as early as fever longer than 3 days. A high sensitivity and odds ratio can provide clinicians with a useful tool for differentiation.

This study has certain limitations. First, this study was a retrospectively reviewed study, and the symptoms and onset of febrile days may have had recall bias from patients and family; furthermore, some medical records did not present complete data. Second, the number of patients diagnosed with KD on the second visit was small, which may weaken the study's statistical power, and we hope to obtain more cases to conduct a larger study in the future. Third, this study has a single center, so our results should be examined in another hospital or managed as a cohort study in the future. Further research is still needed about the clinical factors that can distinguish KD from other febrile diseases.

## Conclusion

Identifying atypical presentations of KD in a timely manner poses a challenge for pediatricians. Delaying treatment can result in cardiovascular sequalae. NLR and CRP are both biomarkers that represent inflammation and immune regulatory pathways and can be used as predictive tools of KD. Among patients suspected of KD that did not initially meet the criteria, clinicians should be aware of an elevated neutrophil-to-lymphocyte ratio, as well as CRP levels, and follow up those patients closely.

## Data Availability Statement

The datasets generated and analyzed during the current study are not publicly available due to strict ethical regulation of information privacy in Taiwan. Requests to access these datasets should be directed to Dr. Ho-Chang Kuo, erickuo48@yahoo.com.tw.

## Ethics Statement

We retrospectively reviewed those cases' medical records, so we did not obtain the informed consent. On the other hand, our study was approved by Institutional Review Board of Chang Gung Memorial Hospital (102-0364B).

## Author Contributions

All authors were involved in drafting the article or revising it critically and all authors approved the final version to be published. MG, Y-HH, and H-CK contributed to conceptualization and study design of the study, J-HY and Y-JL interpreted and statistically analyzed the data, J-HY was the major contributor in writing the manuscript. L-SC and H-CK were responsible for monitoring and data management, reviewing and editing the manuscript.

## Conflict of Interest

The authors declare that the research was conducted in the absence of any commercial or financial relationships that could be construed as a potential conflict of interest.
